# Switching from systemic steroids to high doses of ciclesonide restores the hypothalamic pituitary-adrenal axis

**DOI:** 10.1186/2045-7022-3-S1-P27

**Published:** 2013-05-03

**Authors:** Jerzy Marczak, Maciej Ciebiada, Pawe Górski

**Affiliations:** 1 Medical University of Lodz, Poland; 2 Medical University of Lodz, Department of Pneumonology and Allergy, Poland

## Question

Treatment of difficult asthma with oral corticosteroids (OCS) may suppress hypothalamic-pituitary-adrenal axis. In this study we checked if high doses of ciclesonide instead of OCS may restore the adrenal function without loss of the disease control.

## Methods

In five asthmatics with poor control of the disease despite treatment with systemic corticosteroids OC were replaced with high doses of ciclesonide (1600-2400 µg/day). The pulmonary function tests (PFTs), asthma control test and the morning levels of cortisol and ACTH were measured at baseline and in 28 and 56 day of treatment.

## Results

All patients improved in asthma control scores from mean value 9,4 to, 19,8 in 70 days. In four subjects FEV1 improved significantly with mean increase of up to 585 ml in 70 days ACTH levels were normalized in 3 patients after 28 days of observation and in all patients after 56 days. Cortisol level was normalized in 3 patients after 28 days and in next two subjects after 56 days.

## Conclusions

In patients with difficult to treat asthma switching from the prednisone to high doses of ciclesonide may normalize hypothalamic pituitary adrenal axis function and improves the disease control and PFTs.

